# Ectoparasite load of small mammals in the Serengeti Ecosystem: effects of land use, season, host species, age, sex and breeding status

**DOI:** 10.1007/s00436-022-07439-1

**Published:** 2022-02-05

**Authors:** M. Shilereyo, F. Magige, P. S. Ranke, J. O. Ogutu, E. Røskaft

**Affiliations:** 1 Department of Biology, Norwegian University of Science and Technology (NTNU), Trondheim, Norway; 2 Department of Zoology and Wildlife Conservation, University of Dar es Salaam, Dar es Salaam, Tanzania; 3 Centre for Biodiversity Dynamics, Department of Biology, Norwegian University of Science and Technology (NTNU), Trondheim, Norway; 4 Biostatistics Unit, Institute for Crop Science, University of Hohenheim, Fruwirthstrasse 23, 70599 Stuttgart, Germany

**Keywords:** Small mammals, Ectoparasite load, Serengeti ecosystem, Land use, Rainfall seasonality, Breeding status, Habitat type

## Abstract

Ectoparasite load in small mammals can be influenced by both environmental conditions and host species characteristics. However, the nature of these influences is poorly understood in many ecosystems. We used zero-inflated negative binomial (ZINB) regression models with a log link function to assess variation in ectoparasite load among 19 small mammal host species across different land uses (protection in a park, pastoralism and agriculture), habitat types, seasons, age classes, sexes and breeding statuses. We collected 4258 ectoparasites from 612 individual belonging to 19 different species of small mammals. The average ectoparasite load per individual was higher in the pastoral and agricultural lands than in the National Park. Ectoparasite load varied among species and was the highest for the four common and generalist small mammal species (*Aethomys* sp., *Arvicanthis niloticus*, *Mastomys natalensis*, and *Gerbilliscus vicinus*), most notably in the disturbed pastoral and agricultural lands. It was also higher in the dry than the wet season and for adult males than adult females. These patterns partly reflect the greater mobility of small mammals in the drier conditions; in addition the large body size and home range of males increase the likelihood of encountering parasites. Human disturbance was associated with elevated ectoparasitic load among the small mammals and hence elevated risk of transmission of ectoparasites to humans. As a result, understanding the effect of habitat disturbance on ectoparasite load and its link to zoonotic disease risk should be an important conservation goal and public health priority. Moreover, effective pest control strategies should consider variation in ectoparasite load with land use, habitat type, season and species characteristics.

**Supplementary Information:**

The online version contains supplementary material available at 10.1007/s00436-022-07439-1.

## Introduction

Parasites are becoming increasingly recognized as integral members of ecological communities. They play important roles in, and can adversely affect, populations, communities, and ecosystems in multiple ways (Hugot et al. 2001; Nunn et al. 2003). Parasitic infestation can disrupt community structure by decreasing prey populations, thereby disrupting ecological webs (Utsumi et al. 2010). These effects can modify ecosystem functions, for example, by facilitating grass growth and promoting fires, which suppress trees and can alter carbon cycling in ecosystems (Holdo et al. 2009). However, the effect of parasites in ecosystems depends on the abundance and distribution of the host population.

The distribution of parasites among host individuals within a population is often heterogeneous with a small number of hosts harbouring the majority of parasites (Wilson et al. 2002). Such uneven distribution is caused by host- and parasite-related characteristics and environmental factors that affect host exposure and susceptibility to parasites (Wilson et al. 2002; Poulin 2011). Host-related factors are partly intrinsic and include sex, age, body size and breeding status (Poulin 2011; Viljoen et al. 2011; Postawa and Nagy 2016). Parasitism levels may be sex-biased, with males tending to be more heavily parasitized than females (Matthee et al. 2010). This may be a direct consequence of sexual dimorphism in body size because most mammalian males have larger body sizes than females (Moore and Wilson 2002; Postawa and Szubert-Kruszyńska 2014; Young et al. 2015; Postawa and Nagy 2016). However, other factors include greater mobility and different social contact patterns among males than females (Perez-Orella and Schulte-Hostedde 2005; Krasnov et al. 2012a, b). Moreover, host age may influence parasite load because adults may provide better nutritional resources for parasites than juveniles (Soliman et al. 2001; Fichet-Calvet et al. 2003) and juveniles have had a shorter time to be exposed to ectoparasites. Parasite load is also often higher in breeding than non-breeding individuals because reproduction is associated with increased food acquisition to satisfy elevated nutritional and energetic demands, changes in physiology and, hence, with greater vulnerability to parasites (Folstad and Karter 1992; Hillgarth and Wingfield 1997; Christe et al. 2000; Zhang et al. 2010).

Environmental factors also play important roles in determining the prevalence and intensity of ectoparasites, such that ectoparasite load often covaries with environmental variables such as rainfall and temperature (Poulin 2007). In addition, environmental factors affect the habitats in which the host and its ectoparasites reside (Young et al. 2015). Environmental factors may thus shape ectoparasite abundance directly or indirectly (Krasnov et al. 2002; Pietrock and Marcogliese 2003), either by affecting larvae survival or changing host behaviour and body physiology (Shenbrot et al. 2002; Christe et al. 2007; Young et al. 2017). Environmental seasonality can directly and indirectly affect both the individual hosts (i.e. small mammals) and their ectoparasites (Shenbrot et al. 2002; Young et al. 2017). Ectoparasite prevalence and abundance in small mammals normally peak during the dry season when nutritional and water stresses peak and are usually the lowest in the wet season with minimal nutritional and water stresses (Fichet-Calvet et al. 2003; Viljoen et al. 2011; Buchholz and Dick 2017). Seasonal fluctuations in abundance are thus expected to vary especially among ectoparasite species that spend their time both on and off their hosts such as ticks, mites and fleas (Krasnov et al. 2007; Krasnov 2008).

Furthermore, the variation in ectoparasite load with season and host habitat type can be modified by human land use (Shenbrot et al. 2002; Mize et al. 2011). The abundance of ectoparasites is linked to land use in manifold ways (Krasnov et al. 1997; Mize et al. 2011; Hieronimo et al. 2014). First, land use shapes the distribution of small mammals and their associated ectoparasites (Massawe et al. 2006; Hieronimo et al. 2014). Accordingly, land use can alter the availability, density and susceptibility of individual hosts to ectoparasites (Keesing et al. 2006; Linard et al. 2007; Hieronimo et al. 2014). Second, land use is associated with specific practices that can modify habitat suitability for, and numerical abundance of, small mammals and their ectoparasites. For example, defaunation of biodiversity, particularly of large herbivores and carnivores, has been linked to compositional and functional changes in ecosystems (Bellwood et al. 2003; Keesing and Ostfeld 2021b). This is because large mammals can be important hosts for some ectoparasites and may also regulate the number of small mammals (potential hosts) through competition (Young et al. 2014, 2015). Furthermore, human activities disturb habitats and may favour generalist hosts able to exploit resources from a wide variety of food items and habitats (Molyneux 2003). Thus, changes in susceptible host abundance can represent one pathway through which wildlife loss and disturbance can influence ectoparasite load and rodent-borne disease risk (Young et al. 2014; Khalil et al. 2016). Also, certain practices can disturb habitats and reduce the survival rates of ectoparasites, lowering ectoparasite loads of small mammals (Hieronimo et al. 2014). Activities such as livestock keeping and use of acaricides on livestock may also affect ectoparasite abundance in the environment and reduce the number of ectoparasites available per host. Furthermore, farming can increase the host species through homogenization of habitats, but other activities such as tillage practice can disturb vegetation and soils, reducing habitats for small mammals and their associated ectoparasites (Tavares et al. 2019). Yet, our understanding of ectoparasite load in small mammals and its response to land use, rainfall seasonality and host species’ characteristics is still very limited.

Previous studies of small mammals in the Serengeti (Magige and Senzota 2006; Timbuka and Kabigumila 2006; Byrom et al. 2015) paid relatively little attention to ectoparasites and how they are influenced by human land use, rainfall seasonality and host species’ characteristics. Here, we analyse how ectoparasite load varies with land use, habitat type, environmental seasonality and small mammal host species. More specifically, we evaluate how ectoparasite load varies with species, age (adult vs. juvenile), sex (male vs. female) and breeding status (breeding vs. non-breeding) of the host species under protection in the Serengeti National Park, livestock pastoralism (and limited cultivation) and crop agriculture. We consider 19 small mammal species inhabiting the Serengeti ecosystem numerically dominated by the African grass rat (*Arvicanthis niloticus*) and the multimammate rat (*Mastomys natalensis*) in the wet and dry seasons (Senzota 1982; Shilereyo et al. 2020). We focus on two locally abundant genera of ectoparasites, namely mites (*Echinolaelaps* spp.) and fleas (*Xenopsylla* spp.). Members of the genus *Echinolaelaps* are known to harbour dermatitis- and leptospirosis-causing pathogens (Babolin et al. 2016), while members of the genus *Xenopsylla* are important vectors of the pathogenic bacilli, *Yersinia pestis*, and the agent of murine typhus, *Rickettsia typhi*, that are both harmful to humans and other animals (Dill et al. 2013; Boyer et al. 2014; McCauley et al. 2015; Thomas et al. 2020). Although these two genera exhibit the same life-history strategy of living on and off their hosts, their load is expected to vary with small mammal density (Perez-Orella and Schulte-Hostedde 2005) and to be lower in the protected park than in the disturbed agricultural or pastoral land (Khalil et al. 2016).

Human activities can increase risks and stress leading to low immunity in animals including small mammals (Ezenwa 2004; Martin et al. 2010). This stress can be elevated during the dry season due to food and water shortages (Nunn et al. 2003; Viljoen et al. 2011). Nevertheless, adult male small mammals typically have larger body sizes and often utilize larger home ranges than juveniles and females (Fichet-Calvet et al. 2003; Morand et al. 2004; Krasnov et al. 2012b; Postawa and Nagy 2016). Thus, we test predictions of the following six hypotheses. (H_1_) Ectoparasite load is expected to be higher in the disturbed pastoral and agricultural lands than in the protected park because rodent populations often occur at much higher densities in human-dominated areas, suggesting that some rodent species can cope very well in such areas (Caro 2001; Magige and Senzota 2006; Crespin et al. 2008; Mayamba et al. 2020). (H_2_) Ectoparasite load should be higher in the dry than the wet season regardless of the land use. (H_3_) Adults of both sexes should harbour more ectoparasites than juveniles. (H_4_) Adult males should be infested by more ectoparasites than adult females. (H_5_) Because breeding in mammals exerts high energetic and nutritional demands (Christe et al. 2000), breeding individuals should have more ectoparasites than non-breeding ones (Christe et al. 2000). (H_6_) Finally, because mites and fleas respond differently to rainfall seasonality and have important ecological differences (e.g. mites are more food generalists than fleas (Cruz et al. 2012; Goater et al. 2014)), mites should be more abundant than fleas per individual small mammal host regardless of land use or season (Civitello et al. 2015).

## Materials and methods

### Study area

**Fig.1:**
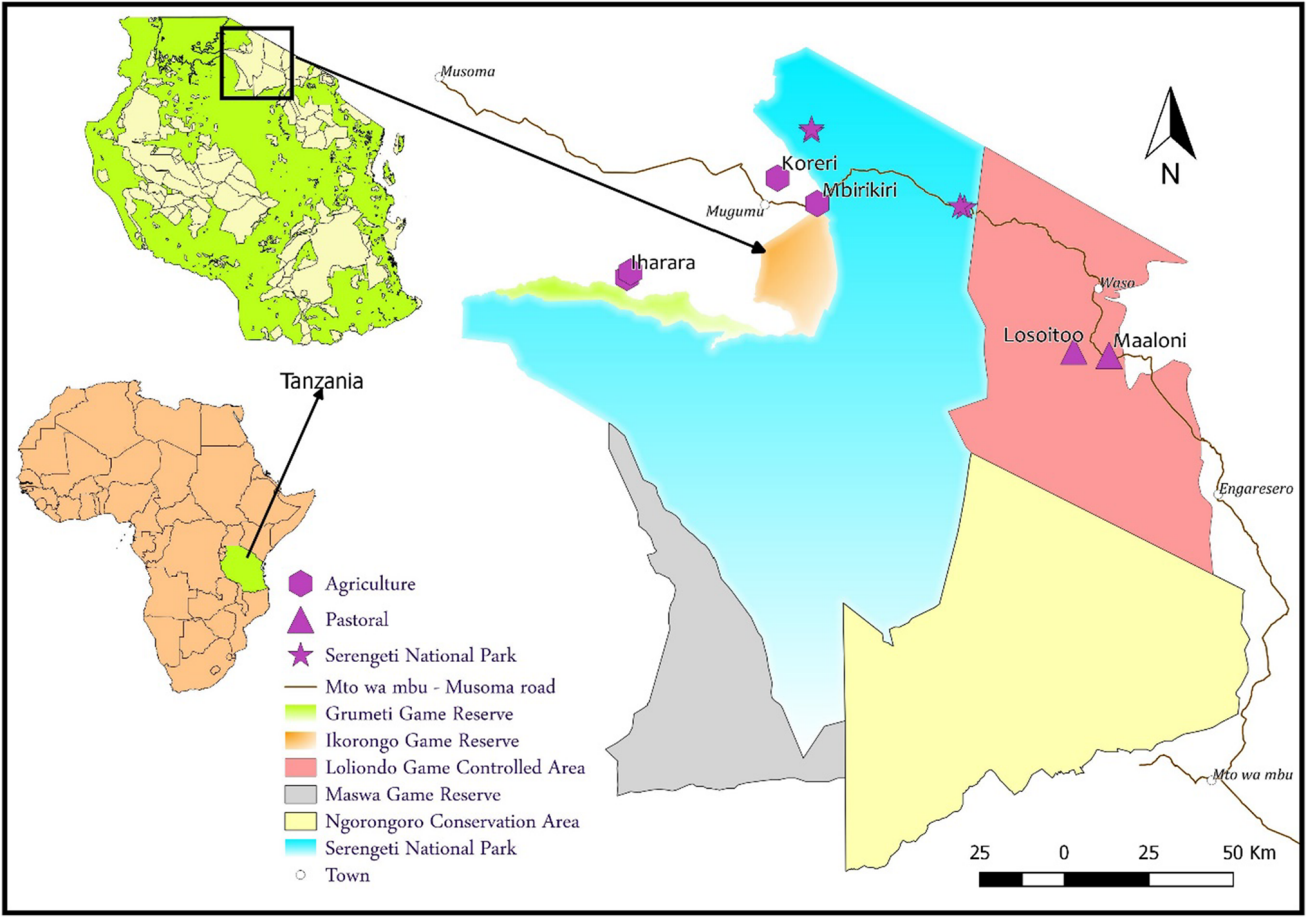
Map of the Serengeti ecosystem showing the study plots (purple colour) arrayed along the Mto wa Mbu-Musoma road transect

The study was conducted in the Serengeti National Park (2° 0′ 0''S and 34° 49′ 60''E) and adjacent villages located in the Ngorongoro (35° 34′ 05'' E and 2° 03′ 47''S) and Serengeti (34° 40′ 26'' E and 1° 50′ 35''S) districts. We used road transects from Mto wa Mbu to Musoma, sampling across three contrasting land uses: pastoral land in the northeastern part of the ecosystem, protected land inside the Serengeti National Park and agricultural land in the western part of the ecosystem (Fig. 1). Within each land use type, four plots were selected based on the dominant habitat types except in the pastoral land where only two plots were selected because of access restrictions due to land conflict between the local villages and the government. The sampling of small mammals was conducted in a total of five habitat types: wooded grassland, shrubland, grassland, riverine forest and cropland. However, riverine forest was only available within the park, whereas cropland was found exclusively in the pastoral and agricultural lands. Thus, trapping was done in a total of 10 habitats belonging to five types, four habitats each in the park and agricultural land and two habitats in the pastoral land. Trapping was done for two consecutive years (2017 and 2018), during the wet and dry seasons.

Rainfall distribution in the ecosystem is bimodal, with the wet season covering October–May and the dry season covering June–September. The wet season can be further partitioned into the short rains (October-December) and the long rains (March–May). January and February are often wet but can be dry in some years, resulting in a short dry season (Norton-Griffiths et al. 1975; Mduma et al. 1999). The climatological year therefore spans from October of the previous to September of the current year.

### Small mammal trapping

A standardized sampling protocol was used in each of the 10 plots. Three trap types were used, namely, Sherman (*n* = 100), bucket pitfalls (*n* = 11) and wire mesh (*n* = 30) traps. Each plot was 100 × 100 m (1 ha) in size, and 100 Sherman traps were set inside and one line of bucket pitfalls at one end of each plot. Pitfall lines consisted of 11 buckets (with a capacity of 20-L each) that were spaced by 5 m. Each of the 11 buckets per line was buried into the ground, 26-cm deep, with the top at the ground level. The pitfall line was connected by 50-cm-high black plastic drift fence running over the centre of the 11 buckets. A similar procedure has been used with success in other small mammal surveys (Stanley et al. 2014) to capture mostly shrews and very small rodents moving on the habitat floor, by following the trail and falling into a bucket (Lyra-Jorge and Pivello 2001; Stanley et al. 2014). Sherman traps were distributed in a line of 10 traps and spaced 10-m apart, and 10 wire mesh traps were placed in line with the Sherman traps in the 3rd, 6th and 9th trapping grid throughout the trapping grid. Shilereyo et al. (2021) provide further details of the layout of traps on the trapping grid. Trapping was conducted for five consecutive nights in each plot before traps were transferred to the next plot. Traps were rebaited daily with a mixture of pieces of freshly fried coconut and Lake Victoria sardines (*Rastrineobola argentea*) coated with peanut butter. This mixture is known to attract a wide array of small mammals (Astúa et al. 2006). Traps were checked twice daily, early in the morning (6:30–8:30 am) and late in the evening (17:00–19:00 pm).

Small mammals were trapped in the wet (April–May) and dry (August–September) seasons of 2017 and 2018. In 2017, trapping was carried out from 18 April to 30 May in the wet season and from 5 August to 25 September in the dry season, while in 2018, we trapped from 10 April to 28 May in the wet season and 5 August to 20 September in the dry season. We started trapping on the pastoral land (eastern part of the ecosystem) followed by the park and the agricultural land (western part of the ecosystem). This order was chosen because the eastern part of the ecosystem dries earlier because it receives less rainfall than the western part. The same sequence was followed at all times except in the wet season of 2018 when logistical difficulties made it necessary to set traps in the park after the agricultural land.

Trapped animals were removed from the traps and put in a cloth bag. Thereafter, they were placed on a white tray, restrained by hand and identified to the genus or species. Each individual was aged (as adult or juvenile) and sexed. Individuals were aged using body size, fur colour and texture and were assigned to adult and juvenile classes. Juveniles have smaller body size and greyer, softer and down-like baby fur and have not undergone reproductive development and other changes associated with adulthood (Searle 1985). By contrast, adults are larger and have different fur colour patterns and texture and fully developed reproductive organs (Searle 1985; Kingdon 2015). Individuals were sexed using external genitalia and secondary sexual characteristics. These included the presence of testes, status of nipples, number of urogenital openings and distance between the anus and urinogenital opening, which is characteristically shorter for female than male rodents (Kunz et al. 1996; Carraway 2009). Sexing of juveniles is often unreliable; thus juveniles were not sexed (Bartolommei et al. 2018). We determined the breeding status of each adult individual by examining the position of the testes—in males (scrotal or abdominal) and for the females, perforated or imperforated. During the breeding season, females have a swollen vaginal tissue and a gaping vaginal opening, whereas males tend to have descended testes due to a temporary bulge in the perineal region (i.e. between the anus and the urinogenital opening). When sex or breeding status or both could not be accurately determined, animals were classed as “undefined” and excluded from the statistical analyses.

Ectoparasites were collected by passing a fine-toothed comb repeatedly (minimum 10 times up and down) over the animal from the base of the ears to the base of the tail to dislodge any parasites onto a tray (Chuluun et al. 2005). The areas around the base of the ear, back of the head and along the spinal cord were also carefully checked for ectoparasites (Bakr and Fagir 2016). The contents of the tray were carefully examined with a hand lens, and any ectoparasite seen was recovered using a sharpened and moistened wooden rod and placed in a collecting tube containing 70% ethanol in individual vials for each host specimen for subsequent identification. Next, each animal was marked by toe-clipping, numbered sequentially and released at the point of capture. Ectoparasites were identified to the genus using morphological features and published taxonomic keys (Schmidt et al. 1977; Smyth and Wakelin 1994; Walker et al. 2014). A light dissecting microscope (Leica) with 40 × magnification was used for identification. For each individual we recorded the total number of fleas, mites and all other ectoparasites. Although it is the most commonly used, our method of searching for ectoparasites implies that only surface parasites could be collected. As a result, the method may exclude burrowing parasites (such as scabies agents) or those that are strongly attached (such as trombiculids) which would require scraping to be collected.

### Statistical data analyses

Ectoparasite abundance is often patchily distributed with many hosts having low parasite intensity, while few hosts have high parasite intensity, often resulting in zero-inflated distributions (Zuur et al. 2010). Accordingly, we used zero-inflated negative binomial (ZINB) regression models, with a log link function to assess the effects on ectoparasite load per individual of land use, habitat type, season, host species, age, sex and breeding status.

Because of small sample sizes and highly uneven distributions, leading to zero ectoparasite or small mammal host counts for some land use types, habitat types or one of the two seasons, we only fitted models to (a) the combined ectoparasite abundance, (b) mite abundance and (c) flea abundance. We thus did not fit ectoparasite- or small mammal species-specific models. We fitted four different models. The first model assessed the total ectoparasite load for adults and juveniles as a function of land use, habitat type, season, age and their interactions. The five levels of habitat type were split, meaning that each level was allowed to enter or leave the model independently of the other levels (Data S1). The second model related the total ectoparasite load for adults only with land use, habitat type, season, sex, breeding status and their interactions (Data S2). The third and fourth models related the total loads of fleas or mites separately to land use, habitat type, season, age and their interactions. All the four models permitted only up to 3-way interactions and used a negative binomial error distribution and a log link function. Further, the total ectoparasite load was offset by the logarithm of relative abundance; the total number of small mammals captured divided by the total trapping effort in each land use or habitat type in the wet, dry or both seasons, to account for variation in abundance relative to trapping effort across the three land uses, habitat types or seasons. Thus, the modelled response variable was total ectoparasite load/(total number of small mammals captured /total trapping effort) (Laudisoit et al. 2009; Hieronimo et al. 2014). Trapping effort was unequal across land use by design because the pastoral land had only two habitat types, whereas the park and agricultural land each had four habitat types. The total number of small mammals trapped also varied across the three land uses, habitat types and seasons. The same fixed effects were included in the count and zero-inflated parts of each two-part ZINB model to examine their particular effects on the presence/absence or abundance of ectoparasites when present.

Model selection was based on information theoretics, specifically the corrected Akaike Information Criterion (AICc) (Burnham and Anderson 2002; Snipes and Taylor 2014). All models imposed a strong hierarchy or marginality constraint, meaning that an interaction term was retained in a model only if its constituent main effects were already in the model. The models were fitted using the SAS HPGENSELECT Procedure (SAS Institute 2020). Significance level was set at α = 0.05 unless stated otherwise.

## Results

Overall, 612 individuals of 19 small mammal species were trapped in the ecosystem during a total of 28,200 trap nights of effort. The two numerically most abundant species in the ecosystem (*Arvicanthis niloticus* and *Mastomys natalensis*) contributed 43% to the total number of captured individuals. Of the captured small mammals, 237 were captured in the park, 277 in the pastoral land and 98 in the agricultural land. The total of 28,200 trap nights of effort consisted of 11,280 trap nights in the park, 11,280 in the pastoral land and 5640 in the agricultural land (for more details see Shilereyo et al. 2020).

The two most abundant small mammal species (*A. niloticus* and *M. natalensis*) contributed 41.1% of all the ectoparasites. Of the 4258 ectoparasites recorded, 3552 were mites (*Echinolaelaps* spp.), 672 were fleas (*Xenopsylla* spp.) and 34 belonged to other genera. Mites, followed by fleas (Figure S4), were therefore the two most common ectoparasite groups among the small mammals. Of the 612 small mammals, 161 had mites only, 28 had fleas only and 60 had both mites and fleas, whereas 363 had neither mites nor fleas.

### Land use and habitat type influences on ectoparasite load

**Fig. 2:**
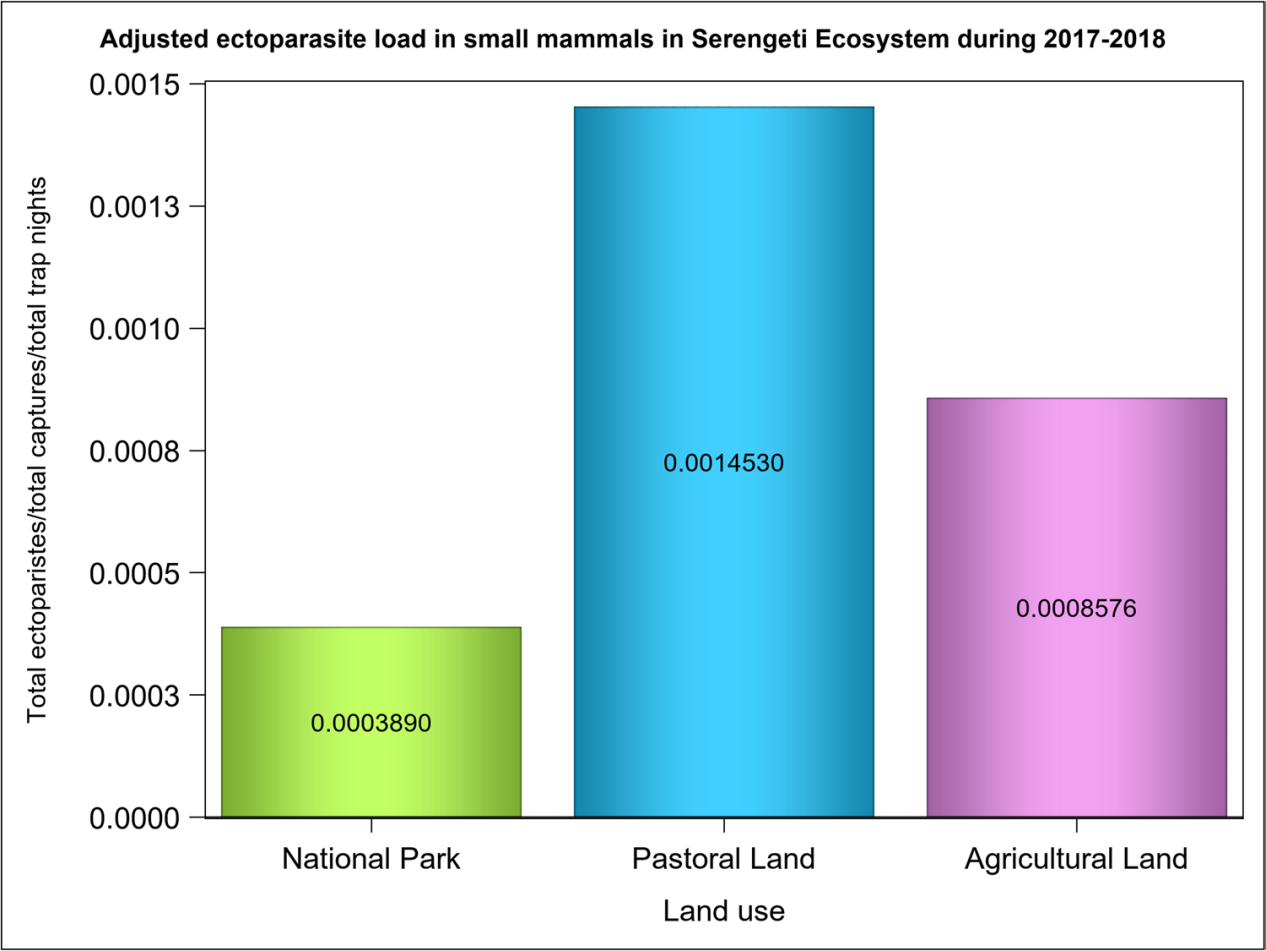
The per individual ectoparasite load of all the small mammals trapped in the park in the Serengeti ecosystem during both the wet and dry seasons of 2017–2018

**Fig. 3:**
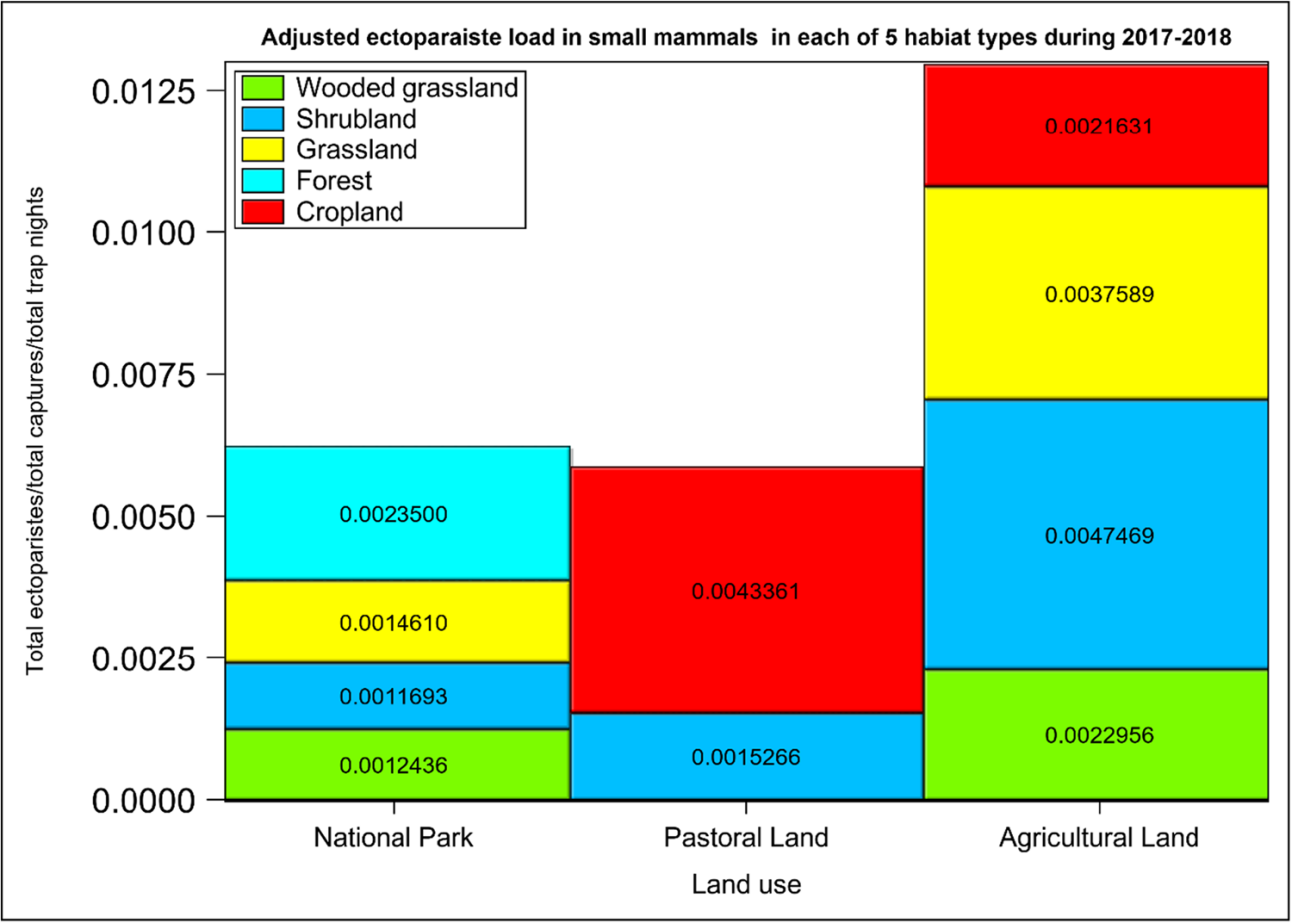
Cumulative (stacked) ectoparasite load per individual small mammal/(total number of captured small mammals/total trap nights) in each of the five habitat types across the three land uses in the Serengeti ecosystem during both the wet and dry seasons of 2017–2018

The average ectoparasite load per individual varied between land uses and habitat types (Figs. 2 and 3). It was the highest in the pastoral land, medium in the agricultural land and the lowest in the park (Fig. 2, Table 1, 0.0003 = [1040 ectoparasites/(237 small mammals/11280 trap nights) = relative abundance]), pastoral land (0.001 = 2270/277/5640) and the agricultural land (0.0008 = 948/98/11280) supporting H_1_. This pattern was slightly different from what was observed in the trap success across the land use and habitats (Figs. S1 and S2). The per individual parasite load for each land use (the total parasite load/(total number of all small mammals captured/total trap nights of effort)), relative to the park, was 2.20 for the agricultural land and 3.74 for the pastoral land. Thus, the disturbed agricultural and pastoral lands had, respectively, twice and thrice as many ectoparasites per individual as the protected park did. Five patterns were evident in the distribution of the average ectoparasite load per individual across the five habitat types, suggesting that human disturbance of habitats elevated ectoparasite load. Specifically, the average ectoparasite load per individual was (i) twice as high in the wooded grassland habitat in the agricultural land as in the park, (ii) fourfold in the shrubland habitat in the agricultural land relative to the park or the pastoral land, (iii) three times higher in the grassland habitat in the agricultural land than in the park and (iv) twice as high in the cropland habitat in the pastoral land as in the agricultural land (Fig. 3). (v) Lastly, it was the highest in the riverine forest and the lowest in the grassland habitat in the park (Fig. 3).

**Table 1:** Parameter estimates using ectoparasite load as the response and land use and age as the independent variables in a zero-inflated negative binomial model to predict the ectoparasite load per individual small mammal trapped in the Serengeti ecosystem during 2017 and 2018

Effect	Land use	Age	Parameter	Level	Estimate	95% LCL	95% UCL	df	SE	${\chi }_{1}^{2}$	*P*
Count model											
Intercept			Intercept		7.60	6.56	8.64	1	0.5	203.82	< 0.001
Land use	Agricultural land		Agricultural land	Agricultural land	1.53	0.81	2.24	1	0.4	17.48	< 0.001
Land use	National park		National park	National park	0.38	− 0.30	1.06	1	0.4	1.19	0.274
Land use	Pastoral land		Pastoral land	Pastoral land	0			0			
Age		Adult	Age adult	Adult	1.57	0.60	2.54	1	0.5	10.02	0.002
Age		Juvenile	Age Juvenile	Juvenile	0			0			
Dispersion			Dispersion		1.19	0.73	1.92	1	0.3		

### Seasonal variation in ectoparasite load

**Fig. 4:**
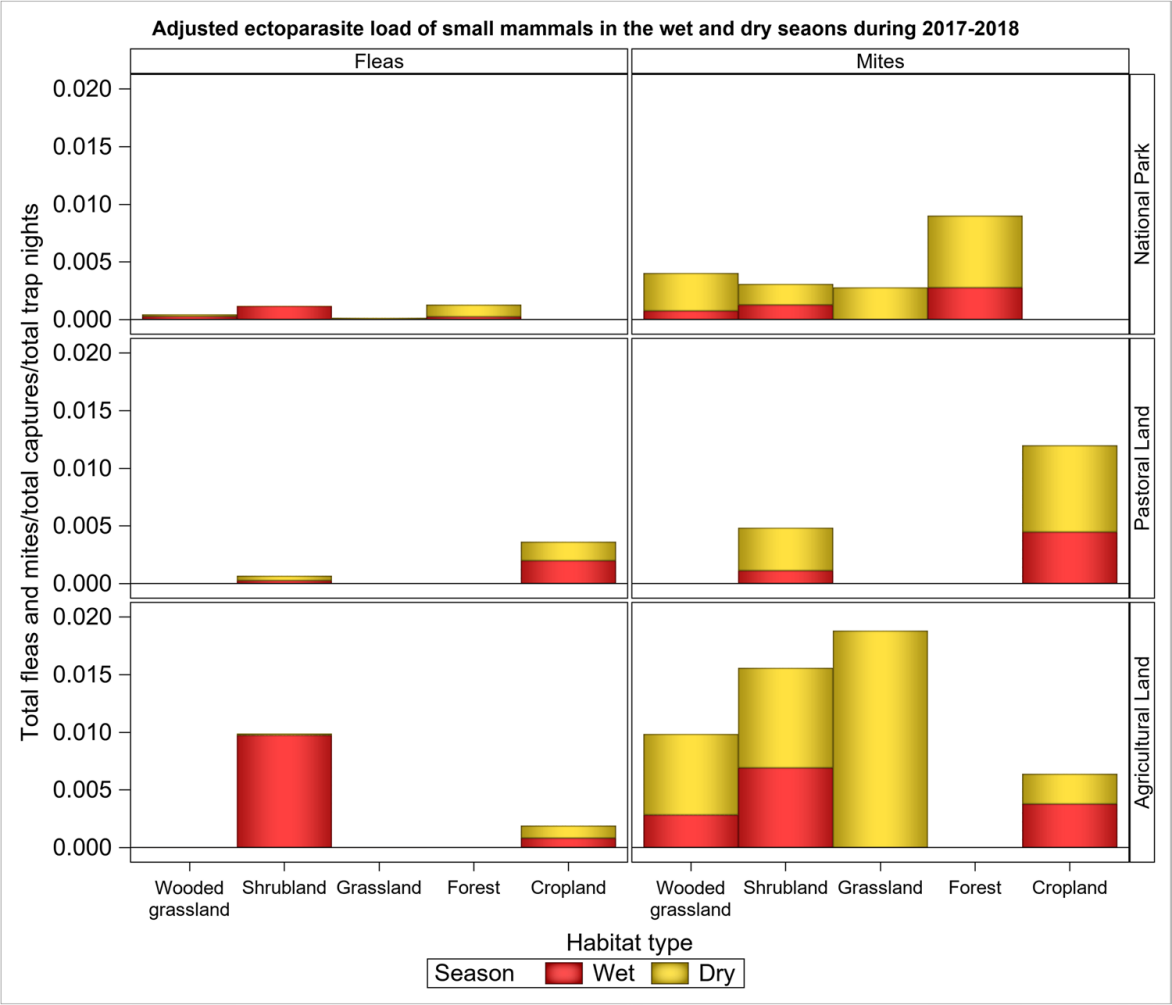
Total ectoparasite load of small mammals/(total captures/total trap night) in each habitat type in the three land uses in the Serengeti ecosystem during the wet and dry seasons of 2017 and 2018

The average ectoparasite load per individual varied between the wet and dry seasons, and the pattern of this seasonal variation differed between the land uses and habitat types (Fig. 4). Generally, ectoparasite load was higher in the dry than the wet season supporting H_2_. Notably, across land uses, the average ectoparasite load per individual was higher in the dry season for all the three land uses, except for the shrubland in the park and shrubland and cropland in the agricultural land, which had higher ectoparasite loads in the wet season (Fig. 4).

**Fig. 5:**
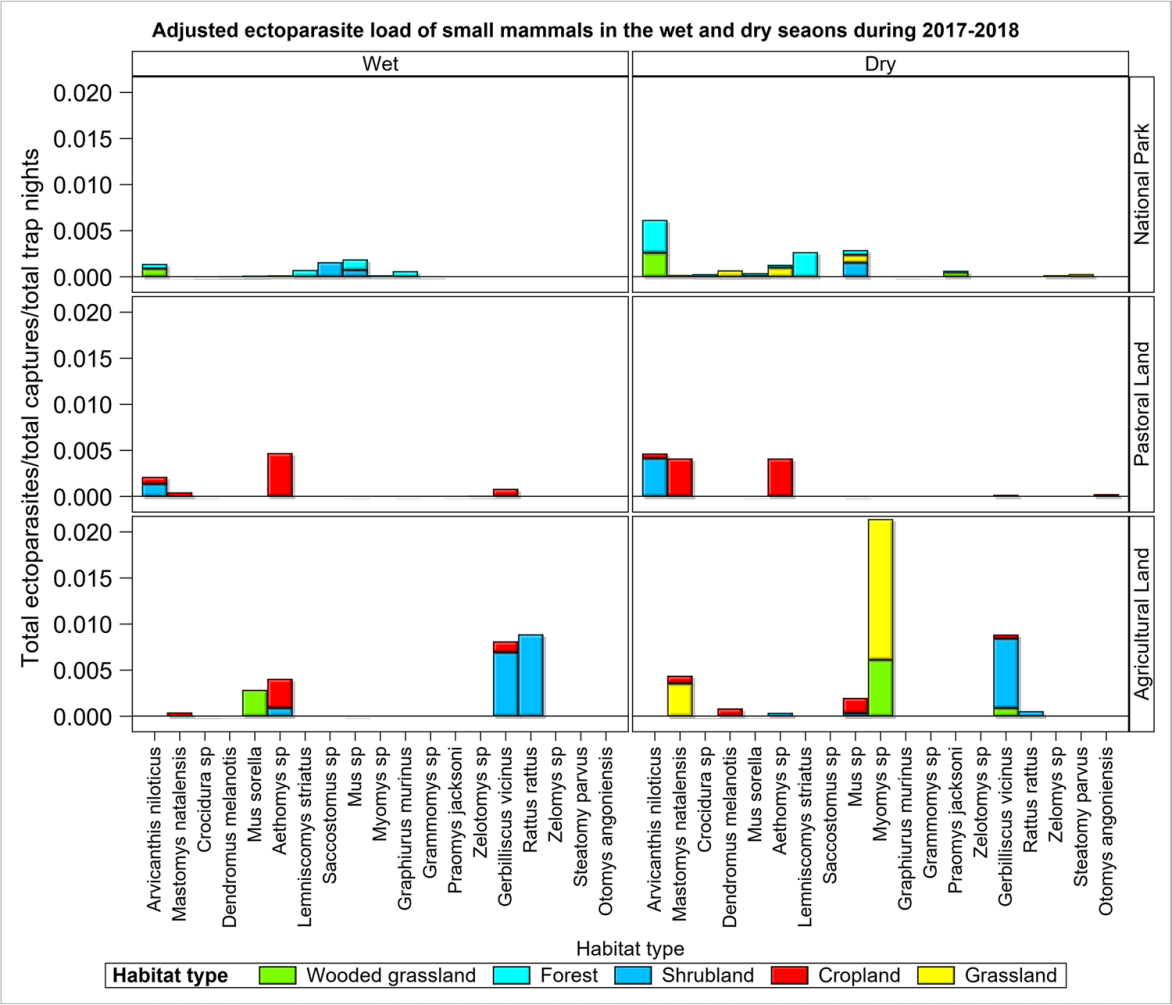
Cumulative (stacked) ectoparasite load of breeding and nonbreeding adult male and female small mammals/total captures for each sex class and breeding status/(total trap nights) in each of the five habitat types and three land uses in the Serengeti ecosystem in the wet and dry seasons of 2017 and 2018

Similarly, the average ectoparasite load per individual varied with the age of the small mammal host (Table 1) and was higher for adults than juveniles as contemplated (H_3_). The variation in the average ectoparasite load with age was apparently independent of season (Table 1, S3 Fig). Furthermore, the average ectoparasite load per individual varied with the sex and breeding status of the adult hosts (Fig. 5). Specifically, adult males had higher average ectoparasite loads per individual than adult females did, as hypothesized (H_4_). The average ectoparasite load per individual was apparently higher for the non-breeding than breeding adults, contrary to expectation (H_5_), and during the dry than the wet season. Season interacted with land use to influence ectoparasite load. As a result, the average ectoparasite load per breeding individual was higher in the dry season in the park and in the wet season in the pastoral land. However, for the agricultural land, seasonal variation in average parasite load was accompanied with variation across habitat types, leading to high loads only in shrubland during the wet season, and especially grassland during the dry season (Fig. 5).

**Fig. 6:**
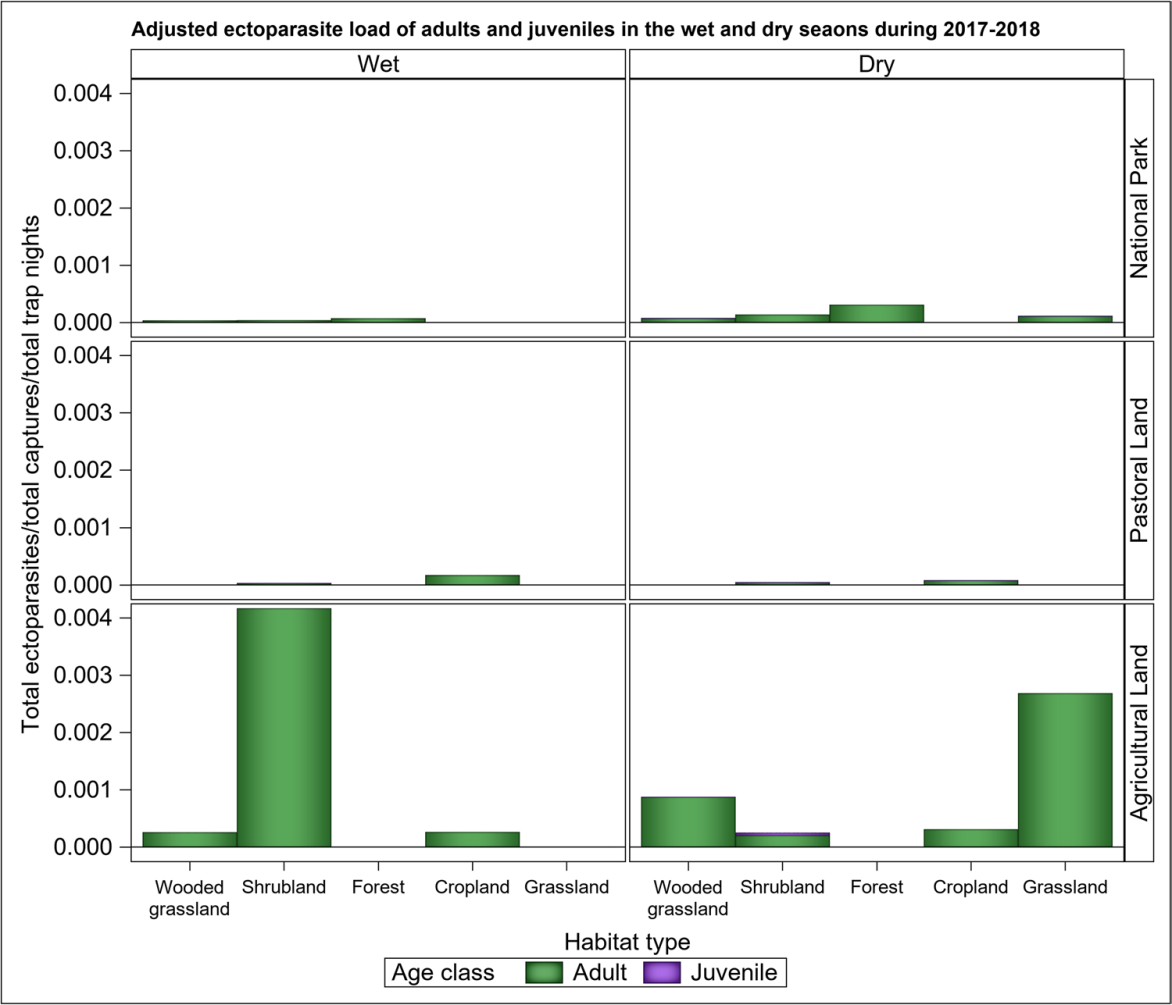
Cumulative (stacked) ectoparasite load per individual for each of the 19 species of small mammals in each of the three land uses in the Serengeti ecosystem during the wet and dry seasons of 2017 and 2018

The average ectoparasite load per individual also varied across host species, and this variation was evidently conditional on land use, habitat type and season (Fig. 6). It was most notably higher in the dry season for one species (*Arvicanthis niloticus*) in the wooded grassland and riverine forest in the park, three species (*Arvicanthis niloticus*, *Mastomys natalensis* and *Aethomys* sp*.*) in the shrubland and cropland in the pastoral land and one species (*Gerbilliscus vicinus*) in the shrubland and another in the grassland (*Myomys* sp. in the agricultural land, consistent with prediction (H_2_).

### Land use, age and seasonal variation in the load of mites and fleas

The average number of mites and fleas per individual varied with small mammal age, season and land use (Tables 2, 3, Tables S1-S2, Fig. S4). For mites it was higher among adults than juveniles, in accord with prediction (H_4_). The average number of mites per individual was also higher during the dry than the wet season and in the pastoral land than in the agricultural land but was comparable between the park and the pastoral land (Tables 2, S3, S4 Fig). The average number of fleas per individual was lower than that for mites and varied little between land uses, habitat types or seasons (Tables 3, S2, Fig. S4). The probability of not being parasitized was also lower in the dry than the wet season (Table 2).

**Table 2:** Parameter estimates (both count and zero parts of the model) of the zero-inflated negative binomial model for the effects of land use, season and age on the mean number of mites (response) per individual small mammal in the Serengeti ecosystem during 2017–2018

Effect	Land use	Season	Age	Parameter	Level	Estimate	95% LCL	95% UCL	df	SE	${\chi }_{1}^{2}$	*P*
Count model												
Intercept				Intercept		6.99	5.88	8.11	1	0.57	150.27	< 0.001
Land use	Agricultural land			Agricultural land	Agricultural land	1.56	0.91	2.22	1	0.33	21.84	< 0.001
Land use	National Park			National park	National park	0.47	− 0.14	1.09	1	0.31	2.31	0.129
Land use	Pastoral land			Pastoral land	Pastoral land	0			0			
Season		Dry		Season dry	Dry	0.56	0.03	1.09	1	0.27	4.24	0.039
Season		Wet		Season wet	Wet	0			0			
Age			Adult	Adult	Adult	1.63	0.71	2.55	1	0.47	11.97	< 0.001
Age			Juvenile	Juvenile	Juvenile	0			0			
Dispersion				Dispersion		0.89	0.59	1.37	1	0.19		
Zero model												
Intercept						− 0.47	− 1.18	0.22	1	0.36	1.79	0.18
Season		Dry				− 1.19	− 2.34	− 0.05	1	0.58344	4.19	0.040

**Table 3:** Parameter estimates of the negative binomial model for the effects of age and habitat type on mean number of fleas (response) per individual small mammal in the Serengeti ecosystem during 2017–2018

Effect	Habitat	Age	Parameter	Level	Estimate	95% LCL	95% UCL	df	SE	${\chi }_{1}^{2}$	*P*
Intercept			Intercept		4.23	1.71	6.76	1	1.28	10.85	≤ 0.001
Habitat	Cropland		Cropland	Cropland	1.55	0.09	3.01	1	0.74	4.38	0.036
Habitat	Forest		Forest	Forest	1.05	− 0.52	2.62	1	0.8	1.71	0.191
Habitat	Grassland		Grassland	Grassland	− 0.39	− 2.39	1.62	1	1.02	0.14	0.705
Habitat	Shrubland		Shrubland	Shrubland	2.61	1.22	4.01	1	0.71	13.46	< 0.001
Habitat	Wooded grassland		Wooded grassland	Wooded grassland	0			0			
Age		Adult	Adult	Adult	2.54	0.39	4.68	1	1.09	5.37	0.020
Age		Juvenile	Juvenile	Juvenile	0			0			
Dispersion			Dispersion		2.19	0.93	5.13	1	0.96		

## Discussion

We analysed variation in ectoparasite load in small mammals among different land uses and habitat types, rainfall seasonality and host species characteristics (age, sex, breeding status). This involved assessing how ectoparasite load varied between land use, habitat type, season and host species and with the age, sex and breeding status of the host species in the Tanzanian Serengeti ecosystem during 2017 and 2018. The average ectoparasite per individual was the highest in the pastoral land, intermediate in the agricultural land and the lowest in the protected park, indicating that human disturbance enhanced ectoparasite load.

Human disturbance apparently increases ectoparasite load per individual most likely by reducing habitat heterogeneity and altering small mammal species diversity. Indeed, the pastoral and agricultural lands supported fewer small mammal species but numerically more abundant than the protected park (Shilereyo et al. 2020). The reduced species diversity in the disturbed pastoral and agricultural lands may increase ectoparasite load (Van Deventer and Nel 2006; Civitello et al. 2015; Peng et al. 2018). This is consistent with the higher average ectoparasite load per individual in the shrubland in the pastoral and agricultural lands and in the grassland and wooded grassland in the agricultural land than in the park. As a result, biodiversity and the spread of ectoparasites may be causally linked. Diverse host communities can lower the abundance of parasites through several mechanisms, such as regulating populations of susceptible hosts, or interfering with the transmission process and reducing the transmission of ectoparasites (Johnson and Thieltges 2010; Keesing et al. 2010; Keesing and Ostfeld 2021a). Thus, diverse communities may suppress the proliferation of parasites, thereby promoting the stability of ecological communities and ecosystem services.

Land use alters habitat quality, microclimate and hence resource availability for some small mammals, leading to higher ectoparasite load. The higher average ectoparasite load per individual in the cropland and shrubland than in the other habitat types most probably reflects the increase in relative abundance of some reservoir small mammal hosts because of available resources in and around these habitats (Laudisoit et al. 2009; Suzán et al. 2009; Peng et al. 2018). This is because both transmission rates and the reproductive success of ectoparasites are often proportional to the abundance of their host (Suzán et al. 2009). However, the high average ectoparasite load per individual in the forest habitat in the park may be related to two possible mechanisms. First, besides habitat quality, host species (e.g. *A. niloticus*) also contributes to the higher average ectoparasite load such as through higher relative abundance in the human-dominated environment. Second, the relatively less disturbed forest floor might provide a conducive microclimate for the ectoparasites during the off-host stage of their life cycle. However, apart from the numerical dominance of certain host species, such as *A. niloticus*, other factors may also play important roles in determining ectoparasite load, including ecological, behavioural, morphological (e.g. interspecific differences in the skin and/or fur) and physiological (e.g. interspecific differences in blood hormonal levels due to stress) characteristics of host species (Hamidi et al. 2015).

The higher ectoparasite load in the dry than the wet season reflects the influence of rainfall seasonality on small mammals, their ectoparasites and environment. Small mammals extend their home ranges during the dry season to satisfy their food and water requirements, thus increasing the probability of contacting parasites. In contrast, during the wet season, the host species aggregate for breeding, which may also facilitate social grooming. Consequently, the results accord with the prediction that ectoparasite load should be higher in the dry than the wet season, corroborating several previous findings (Hamidi et al. 2015; Buchholz and Dick 2017). Seasonality in ectoparasite load may also be caused by seasonal differences in species composition because ectoparasite species may differ in their life histories and strategies.

Seasonality in ectoparasite load portrays distinctions in the responses to rainfall seasonality of different ectoparasite-host species associations. Ectoparasites differ with respect to their associations with host species and thus react differentially to variation in rainfall seasonality. The two numerically most common ectoparasites (mites and fleas) showed equally strong seasonal variation. This reflects their life-history strategy of spending part of their lives off the host, resulting in pronounced seasonality, as expected (Vinarski et al. 2007; Krasnov 2008; Krasnov et al. 2016). Moreover, rainfall seasonality may promote breeding and development in some ectoparasites by interacting with warmth during the dry season to increase breeding success and survival of ectoparasites and hence their likelihood of infesting hosts (Labruna et al. 2009; Peng et al. 2018). Thus, rainfall seasonality and associated changes in temperature can drive changes in the reproductive rates of ectoparasites. As a result, global warming is likely to increase ectoparasites and other animal and human pathogens (Teshome and Girmay 2019). Nevertheless, since we focus on ectoparasite load at the community level, analyses of specific ectoparasite-host species associations would be required to characterize patterns of their seasonal variation, taking into account their contrasting life-history traits and life cycles (Buchholz and Dick 2017).

The relative abundance, variation in the ability to occupy different habitats and habitat specificity (generalist versus specialists) of small mammals are all likely to shape their ectoparasite load. The average ectoparasite load was higher for the numerically more abundant generalist species (*A. niloticus*, *Mastomys natalensis* and *Aethomys sp*.) than for specialist species (*Dendromus melanotis*, *Graphiurus murinus*) in the shrubland and cropland habitats than in the pastoral and agricultural lands, suggesting that ectoparasite load per individual may also increase with increasing host population density, as also reported by Stanko et al. (2002). Because the generalist host species had higher relative abundances in the human-dominated pastoral and agricultural lands, and some ectoparasite species are important vectors for pathogens that cause diseases in domestic animals and humans, it follows that contact with small mammals can potentially increase livestock and human health risks, thus exacerbating human-wildlife conflicts. Not surprisingly, increased abundance of generalist vectors emanating from human activities is strongly associated with increased parasite transmission and incidence of disease outbreaks in both human and wildlife populations (Molyneux 2003). In Africa, *M. natalensis*, *A. niloticus* and *G. vicinus* are well known to be potential pathogen reservoirs of rodent-borne infection (Gratz 1997; Katakweba et al. 2013; Kouadio et al. 2020). Thus, increased abundance of pathogen-carrying hosts increases the likelihood of disease outbreaks. For example, increase in small mammal abundance in Tanzania and Uganda created favourable conditions for epizootic transmission which in turn increased the risk of human plague cases in the region (Kamugisha et al. 2007; Moore et al. 2015).

The age and sex of small mammals influence ectoparasite load. The average ectoparasite load per individual was higher for adult male than adult female hosts and for adult hosts than juveniles. These differences are attributable, at least in part, to the fact that adult male hosts generally have larger body sizes than adult female hosts. Similarly, adult hosts have larger body sizes than juveniles. Therefore, adult hosts provide ectoparasites with larger dimensions and greater availability of niches (Soliman et al. 2001; Korallo et al. 2007; Young et al. 2015) and better nutritional resources than juveniles (Fichet-Calvet et al. 2003). Moreover, larger hosts generally have higher energetic requirements than smaller ones and therefore must travel longer distances in search of food (Perez-Orella and Schulte-Hostedde 2005; Postawa and Nagy 2016). In addition, adult male hosts have larger home ranges than females, which increases their likelihood of encountering ectoparasites (Bergallo and Magnusson 2004; Perez-Orella and Schulte-Hostedde 2005). Thus, because many ectoparasites spend part of their life outside the host (Bush et al. 2001), greater host mobility increases the chance of their infestation by ectoparasites available in the environment or carried by other hosts (Nunn et al. 2003). Additionally, males and females have important differences in physiological make up because immunosuppressive properties of testosterone tend to weaken the body immunity causing a quantifiable decrease in male health (Krasnov et al. 2012b).

Non-breeding individuals had higher average ectoparasite load than breeding individuals contrary to expectation (H_**5**_) and findings of other studies. Breeding individuals in small mammals tend to be more parasitized than non-breeding ones because they allocate more energy to breeding and thus leave less for self-defence against ectoparasites (Wedekind and Folstad 1994; Vandegrift et al. 2008; Krasnov et al. 2012b). However, the lower average ectoparasite load per breeding individual than expected might be due to reduced mobility of females during pregnancy and lactation and hence reduced risk of encountering ectoparasites (Gomez et al. 2011). Moreover, breeding occurs mainly in the wet season when mobility is reduced because of greater water and forage availability, thus also reducing the risk of being infested by ectoparasites (Viljoen et al. 2011). Lastly, breeding hosts may make greater investment in antiparasitic behaviour, such as self-grooming and changing movement patterns to enhance the survival prospects of the breeders and their offspring, an argument that possibly deserves further research.

Although we have focused on only two ectoparasite genera, other genera which were also collected included ticks *Haemaphysalis* spp. and *Rhipicephalus* spp. Furthermore, other flea genera such as *Nosopsyllus* spp. and *Ctenocephalides* spp. were also collected but were too few to analyse. Although fleas rank among the more successful groups of ectoparasites of small mammals (Beaucournu 2004; Oguge et al. 2009), we surprisingly collected relatively fewer fleas than mites. This might partly be due to their lower sociality and greater ability to jump from the host, thus decreasing the probability of their collection. The fact that we collected ectoparasites on an open tray as opposed to Oguge et al. (2009) who used a plastic bag could also explain the lower number in this study. Fleas have a superior jumping ability that helps them both escape from danger and acquire new hosts (Rothschild et al. 1973; Krasnov et al. 2003; Sutton and Burrows 2011). By comparison, flea larvae spend significant time off-host (mainly in burrows or nests), increasing their sensitivity to environmental stressors (Krasnov et al. 2004). Together with the host specificity of some species (Oguge et al. 2009; Goater et al. 2014), these factors might partly explain the relatively low number of fleas. Lastly, although reported to occur extensively in rodents, to be host-specific (Oguge et al. 2009; Lourenço et al. 2020) and to be found in *Mastomys natalensis* and some *Crocidura* spp. (Oguge et al. 2009), lice were surprisingly absent in our relatively small mammal sample.

## Conclusions

In conclusion, land use, habitat type, rainfall seasonality and host characteristics all play important roles in determining ectoparasite load in small mammals in the Serengeti ecosystem. The average ectoparasite load per individual was higher in the disturbed pastoral and agricultural lands suggesting impacts of human activities. The average ectoparasite load per individual was higher in the dry than in the wet season. The age and sex of the host species also influenced the average ectoparasite load, such that it was higher for adults than juveniles and males than females. This suggests that larger body size may provide more nutritional and energetic resources and also that larger home ranges of the host species may increase the risk of infestation. Furthermore, some numerically abundant and generalist species (*Aethomys* sp., *A. niloticus*, *M. natalensis* and *G. vicinus*) had higher per individual ectoparasite load than others, especially in the pastoral and agricultural lands. Consequently, human activities, resulting in increased abundance of generalist small mammals, often pest species, elevate the risk of transmission of zoonotic diseases to humans and livestock and hence human-wildlife conflicts. Rainfall seasonality, land use and habitat and host species’ characteristics should all be considered in designing pest control strategies in human-dominated systems. Maintaining high habitat heterogeneity around protected areas would be necessary to maintain high animal species diversity and minimize ectoparasites load.

## Supplementary Information

Below is the link to the electronic supplementary material.

Supplementary file1 (DOCX 484 KB)

## Data Availability

The datasets generated during and/or analysed during the current study are attached as supplementary materials (Data S1 and S2).
