# Supplementary material for: Ectoparasite load of small mammals in the Serengeti Ecosystem: effects of land use, season, host species, age, sex and breeding status
Source: Parasitol Res. 2022 Feb 5;121(3):823–38. doi: 10.1007/s00436-022-07439-1 (PMC8858283; doi:10.1007/s00436-022-07439-1)
Supplement: Supplementary file 1 — Supplementary file1 (DOCX 484 KB) [file 436_2022_7439_MOESM1_ESM.docx]

SUPPLEMENTARY MATERIALS

**S1 Figure.** Number of individuals captured/100 trap night (trap success) of the small mammals across habitat types in the Serengeti Ecosystem during 2017 and 2018

**S2 Figure**: Number of individuals captured/100 trap nights (trap success) of the small mammals across habitat types in the Serengeti Ecosystem during 2017 and 2018


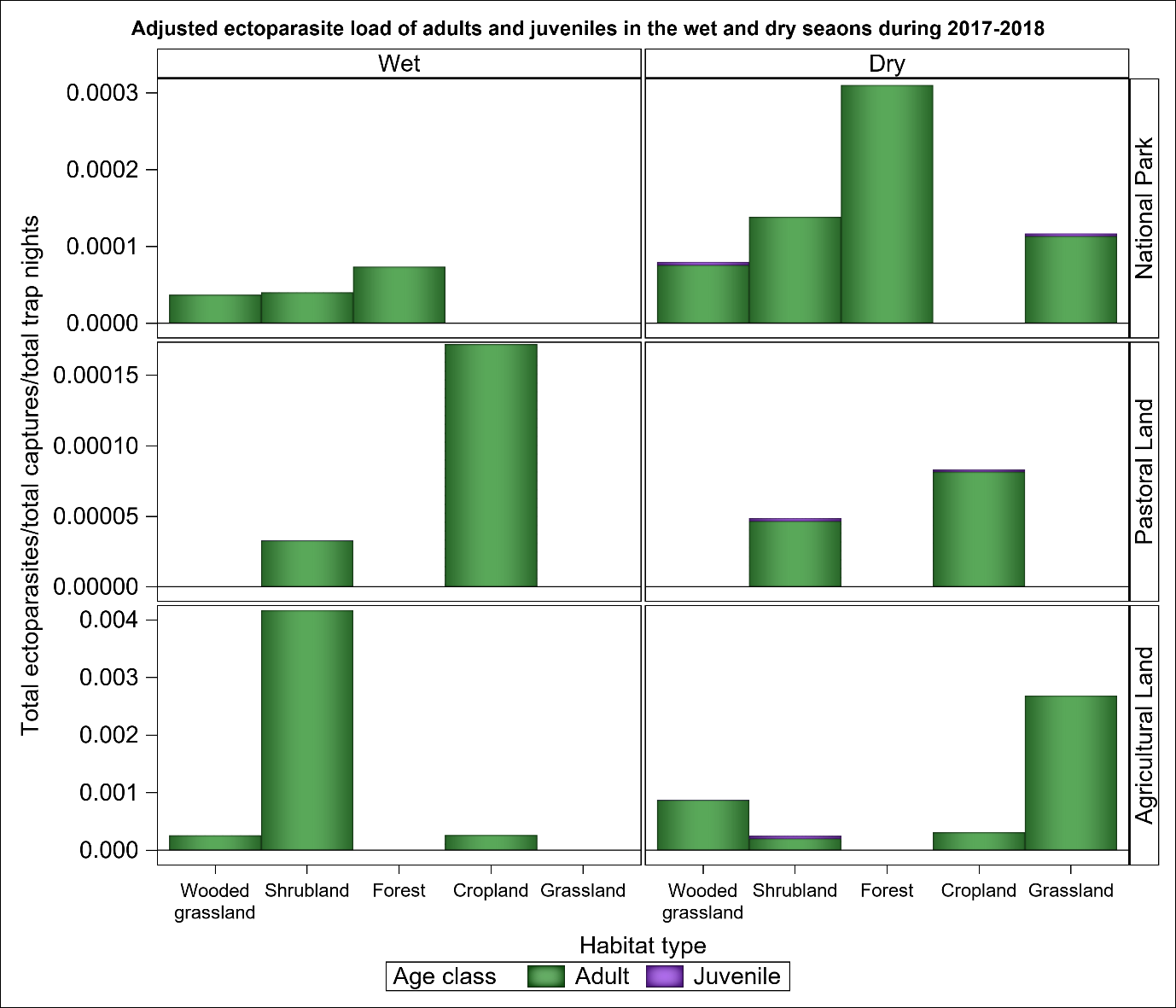


**S3 Figure**. Ectoparasite load of adult and juvenile small mammals in the three land uses and five habitat types in the Serengeti ecosystem in the wet and dry season of 2017 and 2018.


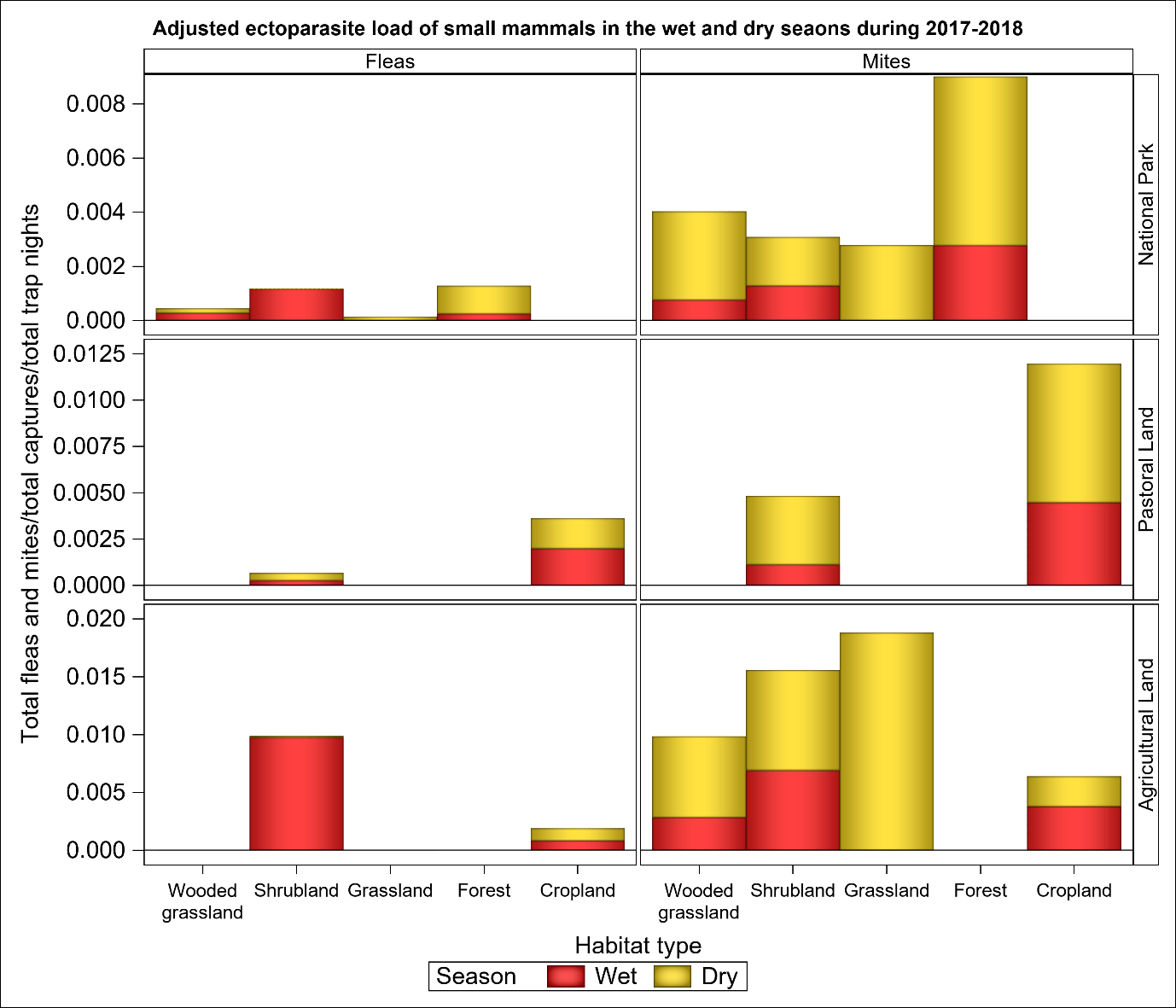


**S4 Figure**. The total number of mites and fleas per individual small mammal/(total captures/total trap night) in the Serengeti ecosystem in the wet and dry season during 2017-2018

**S1 Table**. Parameter estimates of the influence land use on average mite load of small mammal in the Serengeti Ecosystem between 2017-2018.

| Effect | Landuse | Parameter | Level | Estimate | DF | StdErr | ChiSq | ProbChiSq | Lower Confidence Limit | Upper Confidence Limits |
| --- | --- | --- | --- | --- | --- | --- | --- | --- | --- | --- |
| Intercept |  | Intercept |  | 9.1507 | 1 | 0.2375 | 1484.5 | 0 | 8.6852 | 9.6162 |
| Landuse | Agricultural Land | Agricultural land | Agricultural land | 1.293 | 1 | 0.3228 | 16.043 | 6E-05 | 0.6603 | 1.9257 |
| Landuse | National Park | National park | National park | 0.4757 | 1 | 0.31 | 2.3549 | 0.1249 | -0.132 | 1.0832 |
| Landuse | Pastoral Land | Pastoral land | Pastoral land | 0 | 0 |  |  |  |  |  |
| Dispersion | | Dispersion |  | 0.724 | 1 | 0.1589 |  |  | 0.4709 | 1.1131 |

**S2**. Table. Parameter estimates of the influence of habitat on the average number of fleas found on small mammal in the Serengeti Ecosystem during 2017-2018.

| Effect | Habitat | Parameter | Level | Estimate | DF | StdErr | ChiSq | ProbChiSq | Lower Confidence Limits | Upper Confidence Limits |
| --- | --- | --- | --- | --- | --- | --- | --- | --- | --- | --- |
| Intercept |  | Intercept |  | 7.262355 | 1 | 0.701 | 107.3 | 3.86E-25 | 5.8881 | 8.6366 |
| Habitat | Cropland | Cropland | Cropland | 0.963813 | 1 | 0.785 | 1.509 | 0.219259 | -0.574 | 2.5015 |
| Habitat | Forest | Forest | Forest | 0.580268 | 1 | 0.856 | 0.46 | 0.497653 | -1.097 | 2.2572 |
| Habitat | Grassland | Grassland | Grassland | -0.37367 | 1 | 1.28 | 0.085 | 0.770272 | -2.882 | 2.1343 |
| Habitat | Shrubland | Shrubland | Shrubland | 2.326453 | 1 | 0.803 | 8.397 | 0.003757 | 0.7529 | 3.9 |
| Habitat | Wooded grassland | Wooded grassland | Wooded grassland | 0 | 0 |  |  |  |  |  |
| Dispersion | | Dispersion |  | 1.600735 | 1 | 0.441 |  |  | 0.933 | 2.7463 |

**S1 Data**. The distribution of ectoparasite load across land use, habitat type, age, sex and breeding status of small mammals trapped in the Serengeti Ecosystem during wet and dry season of 2017 and 2018.

| **Land use** | **Habitat** | **Season** | **Age** | **Sex** | **Breeding status** | **Parasite load** | **Flea load** | **Mites load** | **Count** | **Effort Land** | **Log effort** |
| --- | --- | --- | --- | --- | --- | --- | --- | --- | --- | --- | --- |
|  |  |  |  |  |  |  |  |  |  | **use** |  |
| Agricultural Land | Cropland | Dry | Adult | Females | Breeding | 25 | 5 | 20 | 12 | 11280 | -6.84588 |
| Agricultural Land | Cropland | Dry | Adult | Females | Not breeding | 0 | 0 | 0 | 12 | 11280 | -6.84588 |
| Agricultural Land | Cropland | Dry | Adult | Males | Breeding | 5 | 5 | 0 | 12 | 11280 | -6.84588 |
| Agricultural Land | Cropland | Dry | Adult | Males | Not breeding | 33 | 8 | 24 | 12 | 11280 | -6.84588 |
| Agricultural Land | Cropland | Wet | Adult | Females | Breeding | 15 | 5 | 10 | 18 | 11280 | -6.44041 |
| Agricultural Land | Cropland | Wet | Adult | Females | Unknown | 0 | 0 | 0 | 18 | 11280 | -6.44041 |
| Agricultural Land | Cropland | Wet | Adult | Males | Breeding | 95 | 15 | 80 | 18 | 11280 | -6.44041 |
| Agricultural Land | Cropland | Wet | Adult | Males | Not breeding | 10 | 1 | 6 | 18 | 11280 | -6.44041 |
| Agricultural Land | Grassland | Dry | Adult | Females | Breeding | 0 | 0 | 0 | 7 | 11280 | -7.38488 |
| Agricultural Land | Grassland | Dry | Adult | Males | Breeding | 13 | 0 | 13 | 7 | 11280 | -7.38488 |
| Agricultural Land | Grassland | Dry | Adult | Males | Not breeding | 40 | 0 | 40 | 7 | 11280 | -7.38488 |
| Agricultural Land | Grassland | Wet | Adult | Males | Not breeding | 0 | 0 | 0 | 3 | 11280 | -8.23217 |
| Agricultural Land | Grassland | Wet | Adult | Unknown | Unknown | 0 | 0 | 0 | 3 | 11280 | -8.23217 |
| Agricultural Land | Shrubland | Dry | Adult | Females | Breeding | 100 | 0 | 100 | 35 | 11280 | -5.77544 |
| Agricultural Land | Shrubland | Dry | Adult | Males | Breeding | 110 | 0 | 110 | 35 | 11280 | -5.77544 |
| Agricultural Land | Shrubland | Dry | Adult | Males | Not breeding | 195 | 7 | 188 | 35 | 11280 | -5.77544 |
| Agricultural Land | Shrubland | Dry | Juvenile | NA | Juvenile | 90 | 0 | 90 | 35 | 11280 | -5.77544 |
| Agricultural Land | Shrubland | Wet | Adult | Females | Not breeding | 35 | 10 | 25 | 4 | 11280 | -7.94449 |
| Agricultural Land | Shrubland | Wet | Adult | Males | Not breeding | 59 | 45 | 14 | 4 | 11280 | -7.94449 |
| Agricultural Land | Wooded grassland | Dry | Adult | Females | Not breeding | 60 | 0 | 60 | 8 | 11280 | -7.25134 |
| Agricultural Land | Wooded grassland | Dry | Adult | Males | Breeding | 10 | 0 | 10 | 8 | 11280 | -7.25134 |
| Agricultural Land | Wooded grassland | Dry | Adult | Males | Not breeding | 9 | 0 | 9 | 8 | 11280 | -7.25134 |
| Agricultural Land | Wooded grassland | Dry | Juvenile | NA | Juvenile | 0 | 0 | 0 | 8 | 11280 | -7.25134 |
| Agricultural Land | Wooded grassland | Wet | Adult | Males | Breeding | 0 | 0 | 0 | 11 | 11280 | -6.93289 |
| Agricultural Land | Wooded grassland | Wet | Adult | Males | Not breeding | 29 | 0 | 29 | 11 | 11280 | -6.93289 |
| Agricultural Land | Wooded grassland | Wet | Adult | Unknown | Unknown | 15 | 0 | 15 | 11 | 11280 | -6.93289 |
| National Park | Forest | Dry | Adult | Females | Breeding | 80 | 15 | 65 | 24 | 11280 | -6.15273 |
| National Park | Forest | Dry | Adult | Females | Not breeding | 5 | 0 | 5 | 24 | 11280 | -6.15273 |
| National Park | Forest | Dry | Adult | Males | Breeding | 120 | 10 | 110 | 24 | 11280 | -6.15273 |
| National Park | Forest | Dry | Adult | Males | Not breeding | 47 | 10 | 31 | 24 | 11280 | -6.15273 |
| National Park | Forest | Dry | Adult | Unknown | Unknown | 0 | 0 | 0 | 24 | 11280 | -6.15273 |
| National Park | Forest | Wet | Adult | Females | Breeding | 59 | 5 | 48 | 43 | 11280 | -5.56959 |
| National Park | Forest | Wet | Adult | Females | Not breeding | 0 | 0 | 0 | 43 | 11280 | -5.56959 |
| National Park | Forest | Wet | Adult | Females | Unknown | 3 | 0 | 3 | 43 | 11280 | -5.56959 |
| National Park | Forest | Wet | Adult | Males | Breeding | 45 | 7 | 38 | 43 | 11280 | -5.56959 |
| National Park | Forest | Wet | Adult | Males | Not breeding | 78 | 3 | 72 | 43 | 11280 | -5.56959 |
| National Park | Forest | Wet | Adult | Males | Unknown | 7 | 0 | 7 | 43 | 11280 | -5.56959 |
| National Park | Forest | Wet | Adult | Unknown | Unknown | 0 | 0 | 0 | 43 | 11280 | -5.56959 |
| National Park | Grassland | Dry | Adult | Females | Not breeding | 79 | 5 | 74 | 25 | 11054.4 | -6.09171 |
| National Park | Grassland | Dry | Adult | Males | Not breeding | 21 | 0 | 21 | 25 | 11054.4 | -6.09171 |
| National Park | Grassland | Dry | Adult | Unknown | Unknown | 0 | 0 | 0 | 25 | 11054.4 | -6.09171 |
| National Park | Grassland | Dry | Juvenile | NA | Juvenile | 3 | 0 | 3 | 25 | 11054.4 | -6.09171 |
| National Park | Shrubland | Dry | Adult | Females | Breeding | 20 | 0 | 20 | 13 | 11280 | -6.76584 |
| National Park | Shrubland | Dry | Adult | Females | Not breeding | 0 | 0 | 0 | 13 | 11280 | -6.76584 |
| National Park | Shrubland | Dry | Adult | Males | Breeding | 0 | 0 | 0 | 13 | 11280 | -6.76584 |
| National Park | Shrubland | Dry | Adult | Males | Not breeding | 13 | 0 | 13 | 13 | 11280 | -6.76584 |
| National Park | Shrubland | Wet | Adult | Females | Breeding | 60 | 20 | 40 | 61 | 11280 | -5.21991 |
| National Park | Shrubland | Wet | Adult | Females | Not breeding | 15 | 5 | 10 | 61 | 11280 | -5.21991 |
| National Park | Shrubland | Wet | Adult | Males | Breeding | 15 | 15 | 0 | 61 | 11280 | -5.21991 |
| National Park | Shrubland | Wet | Adult | Males | Not breeding | 121 | 61 | 60 | 61 | 11280 | -5.21991 |
| National Park | Shrubland | Wet | Adult | Unknown | Unknown | 0 | 0 | 0 | 61 | 11280 | -5.21991 |
| National Park | Wooded grassland | Dry | Adult | Females | Not breeding | 50 | 0 | 50 | 43 | 11280 | -5.56959 |
| National Park | Wooded grassland | Dry | Adult | Males | Breeding | 60 | 10 | 50 | 43 | 11280 | -5.56959 |
| National Park | Wooded grassland | Dry | Adult | Males | Not breeding | 88 | 0 | 88 | 43 | 11280 | -5.56959 |
| National Park | Wooded grassland | Dry | Juvenile | NA | Juvenile | 10 | 0 | 10 | 43 | 11280 | -5.56959 |
| National Park | Wooded grassland | Wet | Adult | Females | Breeding | 35 | 10 | 25 | 28 | 11280 | -5.99858 |
| National Park | Wooded grassland | Wet | Adult | Males | Breeding | 6 | 1 | 5 | 28 | 11280 | -5.99858 |
| National Park | Wooded grassland | Wet | Adult | Males | Not breeding | 0 | 0 | 0 | 28 | 11280 | -5.99858 |
| National Park | Wooded grassland | Wet | Adult | Males | Unknown | 0 | 0 | 0 | 28 | 11280 | -5.99858 |
| National Park | Wooded grassland | Wet | Adult | Unknown | Unknown | 0 | 0 | 0 | 28 | 11280 | -5.99858 |
| Pastoral Land | Cropland | Dry | Adult | Females | Breeding | 482 | 97 | 385 | 110 | 5640 | -3.93716 |
| Pastoral Land | Cropland | Dry | Adult | Females | Not breeding | 45 | 9 | 35 | 110 | 5640 | -3.93716 |
| Pastoral Land | Cropland | Dry | Adult | Males | Breeding | 551 | 18 | 533 | 110 | 5640 | -3.93716 |
| Pastoral Land | Cropland | Dry | Adult | Males | Not breeding | 312 | 112 | 200 | 110 | 5640 | -3.93716 |
| Pastoral Land | Cropland | Dry | Adult | NA | Unknown | 2 | 0 | 2 | 110 | 5640 | -3.93716 |
| Pastoral Land | Cropland | Dry | Juvenile | NA | Juvenile | 25 | 16 | 9 | 110 | 5640 | -3.93716 |
| Pastoral Land | Cropland | Wet | Adult | Females | Breeding | 49 | 6 | 43 | 39 | 5640 | -4.97408 |
| Pastoral Land | Cropland | Wet | Adult | Males | Breeding | 88 | 25 | 63 | 39 | 5640 | -4.97408 |
| Pastoral Land | Cropland | Wet | Adult | Males | Not breeding | 109 | 42 | 58 | 39 | 5640 | -4.97408 |
| Pastoral Land | Cropland | Wet | Adult | Unknown | Unknown | 0 | 0 | 0 | 39 | 5640 | -4.97408 |
| Pastoral Land | Shrubland | Dry | Adult | Females | Breeding | 7 | 4 | 3 | 85 | 5640 | -4.19499 |
| Pastoral Land | Shrubland | Dry | Adult | Females | Not breeding | 106 | 0 | 106 | 85 | 5640 | -4.19499 |
| Pastoral Land | Shrubland | Dry | Adult | Males | Breeding | 5 | 0 | 5 | 85 | 5640 | -4.19499 |
| Pastoral Land | Shrubland | Dry | Adult | Males | Not breeding | 357 | 44 | 310 | 85 | 5640 | -4.19499 |
| Pastoral Land | Shrubland | Dry | Juvenile | NA | Juvenile | 20 | 0 | 20 | 85 | 5640 | -4.19499 |
| Pastoral Land | Shrubland | Wet | Adult | Females | Breeding | 51 | 11 | 40 | 43 | 5640 | -4.87644 |
| Pastoral Land | Shrubland | Wet | Adult | Females | Not breeding | 1 | 0 | 0 | 43 | 5640 | -4.87644 |
| Pastoral Land | Shrubland | Wet | Adult | Males | Breeding | 0 | 0 | 0 | 43 | 5640 | -4.87644 |
| Pastoral Land | Shrubland | Wet | Adult | Males | Not breeding | 60 | 10 | 49 | 43 | 5640 | -4.87644 |
| Pastoral Land | Shrubland | Wet | Juvenile | NA | Juvenile | 0 | 0 | 0 | 43 | 5640 | -4.87644 |

**S2 Data**. The distribution of ectoparasite load across land use, habitat type, season, age, sex and breeding status of small mammals trapped in the Serengeti Ecosystem during wet and dry season between 2017 and 2018.

| **Land use** | **Habitat** | **Season** | **Age** | **Sex** | **Breeding status** | **Parasite load** | **Flea load** | **Mites load** | **Count** | **Effort Land**  **use** | **Log effort** |
| --- | --- | --- | --- | --- | --- | --- | --- | --- | --- | --- | --- |
| Agricultural Land | Cropland | Dry | Adult | Females | Breeding | 25 | 5 | 20 | 12 | 11280 | -6.84588 |
| Agricultural Land | Cropland | Dry | Adult | Females | Not breeding | 0 | 0 | 0 | 12 | 11280 | -6.84588 |
| Agricultural Land | Cropland | Dry | Adult | Males | Breeding | 5 | 5 | 0 | 12 | 11280 | -6.84588 |
| Agricultural Land | Cropland | Dry | Adult | Males | Not breeding | 33 | 8 | 24 | 12 | 11280 | -6.84588 |
| Agricultural Land | Cropland | Wet | Adult | Females | Breeding | 15 | 5 | 10 | 18 | 11280 | -6.44041 |
| Agricultural Land | Cropland | Wet | Adult | Males | Breeding | 95 | 15 | 80 | 18 | 11280 | -6.44041 |
| Agricultural Land | Cropland | Wet | Adult | Males | Not breeding | 10 | 1 | 6 | 18 | 11280 | -6.44041 |
| Agricultural Land | Grassland | Dry | Adult | Females | Breeding | 0 | 0 | 0 | 7 | 11280 | -7.38488 |
| Agricultural Land | Grassland | Dry | Adult | Males | Breeding | 13 | 0 | 13 | 7 | 11280 | -7.38488 |
| Agricultural Land | Grassland | Dry | Adult | Males | Not breeding | 40 | 0 | 40 | 7 | 11280 | -7.38488 |
| Agricultural Land | Grassland | Wet | Adult | Males | Not breeding | 0 | 0 | 0 | 3 | 11280 | -8.23217 |
| Agricultural Land | Shrubland | Dry | Adult | Females | Breeding | 100 | 0 | 100 | 35 | 11280 | -5.77544 |
| Agricultural Land | Shrubland | Dry | Adult | Males | Breeding | 110 | 0 | 110 | 35 | 11280 | -5.77544 |
| Agricultural Land | Shrubland | Dry | Adult | Males | Not breeding | 195 | 7 | 188 | 35 | 11280 | -5.77544 |
| Agricultural Land | Shrubland | Wet | Adult | Females | Not breeding | 35 | 10 | 25 | 4 | 11280 | -7.94449 |
| Agricultural Land | Shrubland | Wet | Adult | Males | Not breeding | 59 | 45 | 14 | 4 | 11280 | -7.94449 |
| Agricultural Land | Wooded grassland | Dry | Adult | Females | Not breeding | 60 | 0 | 60 | 8 | 11280 | -7.25134 |
| Agricultural Land | Wooded grassland | Dry | Adult | Males | Breeding | 10 | 0 | 10 | 8 | 11280 | -7.25134 |
| Agricultural Land | Wooded grassland | Dry | Adult | Males | Not breeding | 9 | 0 | 9 | 8 | 11280 | -7.25134 |
| Agricultural Land | Wooded grassland | Wet | Adult | Males | Breeding | 0 | 0 | 0 | 11 | 11280 | -6.93289 |
| Agricultural Land | Wooded grassland | Wet | Adult | Males | Not breeding | 29 | 0 | 29 | 11 | 11280 | -6.93289 |
| National Park | Forest | Dry | Adult | Females | Breeding | 80 | 15 | 65 | 24 | 11280 | -6.15273 |
| National Park | Forest | Dry | Adult | Females | Not breeding | 5 | 0 | 5 | 24 | 11280 | -6.15273 |
| National Park | Forest | Dry | Adult | Males | Breeding | 120 | 10 | 110 | 24 | 11280 | -6.15273 |
| National Park | Forest | Dry | Adult | Males | Not breeding | 47 | 10 | 31 | 24 | 11280 | -6.15273 |
| National Park | Forest | Wet | Adult | Females | Breeding | 59 | 5 | 48 | 43 | 11280 | -5.56959 |
| National Park | Forest | Wet | Adult | Females | Not breeding | 0 | 0 | 0 | 43 | 11280 | -5.56959 |
| National Park | Forest | Wet | Adult | Males | Breeding | 45 | 7 | 38 | 43 | 11280 | -5.56959 |
| National Park | Forest | Wet | Adult | Males | Not breeding | 78 | 3 | 72 | 43 | 11280 | -5.56959 |
| National Park | Grassland | Dry | Adult | Females | Not breeding | 79 | 5 | 74 | 25 | 11054.4 | -6.09171 |
| National Park | Grassland | Dry | Adult | Males | Not breeding | 21 | 0 | 21 | 25 | 11054.4 | -6.09171 |
| National Park | Shrubland | Dry | Adult | Females | Breeding | 20 | 0 | 20 | 13 | 11280 | -6.76584 |
| National Park | Shrubland | Dry | Adult | Females | Not breeding | 0 | 0 | 0 | 13 | 11280 | -6.76584 |
| National Park | Shrubland | Dry | Adult | Males | Breeding | 0 | 0 | 0 | 13 | 11280 | -6.76584 |
| National Park | Shrubland | Dry | Adult | Males | Not breeding | 13 | 0 | 13 | 13 | 11280 | -6.76584 |
| National Park | Shrubland | Wet | Adult | Females | Breeding | 60 | 20 | 40 | 61 | 11280 | -5.21991 |
| National Park | Shrubland | Wet | Adult | Females | Not breeding | 15 | 5 | 10 | 61 | 11280 | -5.21991 |
| National Park | Shrubland | Wet | Adult | Males | Breeding | 15 | 15 | 0 | 61 | 11280 | -5.21991 |
| National Park | Shrubland | Wet | Adult | Males | Not breeding | 121 | 61 | 60 | 61 | 11280 | -5.21991 |
| National Park | Wooded grassland | Dry | Adult | Females | Not breeding | 50 | 0 | 50 | 43 | 11280 | -5.56959 |
| National Park | Wooded grassland | Dry | Adult | Males | Breeding | 60 | 10 | 50 | 43 | 11280 | -5.56959 |
| National Park | Wooded grassland | Dry | Adult | Males | Not breeding | 88 | 0 | 88 | 43 | 11280 | -5.56959 |
| National Park | Wooded grassland | Wet | Adult | Females | Breeding | 35 | 10 | 25 | 28 | 11280 | -5.99858 |
| National Park | Wooded grassland | Wet | Adult | Males | Breeding | 6 | 1 | 5 | 28 | 11280 | -5.99858 |
| National Park | Wooded grassland | Wet | Adult | Males | Not breeding | 0 | 0 | 0 | 28 | 11280 | -5.99858 |
| Pastoral Land | Cropland | Dry | Adult | Females | Breeding | 482 | 97 | 385 | 110 | 5640 | -3.93716 |
| Pastoral Land | Cropland | Dry | Adult | Females | Not breeding | 45 | 9 | 35 | 110 | 5640 | -3.93716 |
| Pastoral Land | Cropland | Dry | Adult | Males | Breeding | 551 | 18 | 533 | 110 | 5640 | -3.93716 |
| Pastoral Land | Cropland | Dry | Adult | Males | Not breeding | 312 | 112 | 200 | 110 | 5640 | -3.93716 |
| Pastoral Land | Cropland | Wet | Adult | Females | Breeding | 49 | 6 | 43 | 39 | 5640 | -4.97408 |
| Pastoral Land | Cropland | Wet | Adult | Males | Breeding | 88 | 25 | 63 | 39 | 5640 | -4.97408 |
| Pastoral Land | Cropland | Wet | Adult | Males | Not breeding | 109 | 42 | 58 | 39 | 5640 | -4.97408 |
| Pastoral Land | Shrubland | Dry | Adult | Females | Breeding | 7 | 4 | 3 | 85 | 5640 | -4.19499 |
| Pastoral Land | Shrubland | Dry | Adult | Females | Not breeding | 106 | 0 | 106 | 85 | 5640 | -4.19499 |
| Pastoral Land | Shrubland | Dry | Adult | Males | Breeding | 5 | 0 | 5 | 85 | 5640 | -4.19499 |
| Pastoral Land | Shrubland | Dry | Adult | Males | Not breeding | 357 | 44 | 310 | 85 | 5640 | -4.19499 |
| Pastoral Land | Shrubland | Wet | Adult | Females | Breeding | 51 | 11 | 40 | 43 | 5640 | -4.87644 |
| Pastoral Land | Shrubland | Wet | Adult | Females | Not breeding | 1 | 0 | 0 | 43 | 5640 | -4.87644 |
| Pastoral Land | Shrubland | Wet | Adult | Males | Breeding | 0 | 0 | 0 | 43 | 5640 | -4.87644 |
| Pastoral Land | Shrubland | Wet | Adult | Males | Not breeding | 60 | 10 | 49 | 43 | 5640 | -4.87644 |
